# Supplementary material for: The Interfascicular Matrix of Energy Storing Tendons Houses Heterogenous Cell Populations Disproportionately Affected by Aging
Source: Aging Dis. 2024 Feb 1;15(1):295–310. doi: 10.14336/AD.2023.0425-1 (PMC10796100; doi:10.14336/AD.2023.0425-1)
Supplement: Supplementary file 1 [file AD-15-1-295-s.pdf]

SUPPLEMENTARY DATA

The Interfascicular Matrix of Energy Storing Tendons Houses Heterogenous Cell Populations Disproportionately Affected by Aging

Danae E. Zamboulis, Neil Marr, Luca Lenzi, Helen L. Birch, Hazel R. C. Screen, Peter D. Clegg, Chavaunne T. Thorpe

© 2023. Zamboulis DE et al. Published online at http://www.aginganddisease.org/EN/10.14336/AD.2023.0425-1

SUPPLEMENTARY DATA

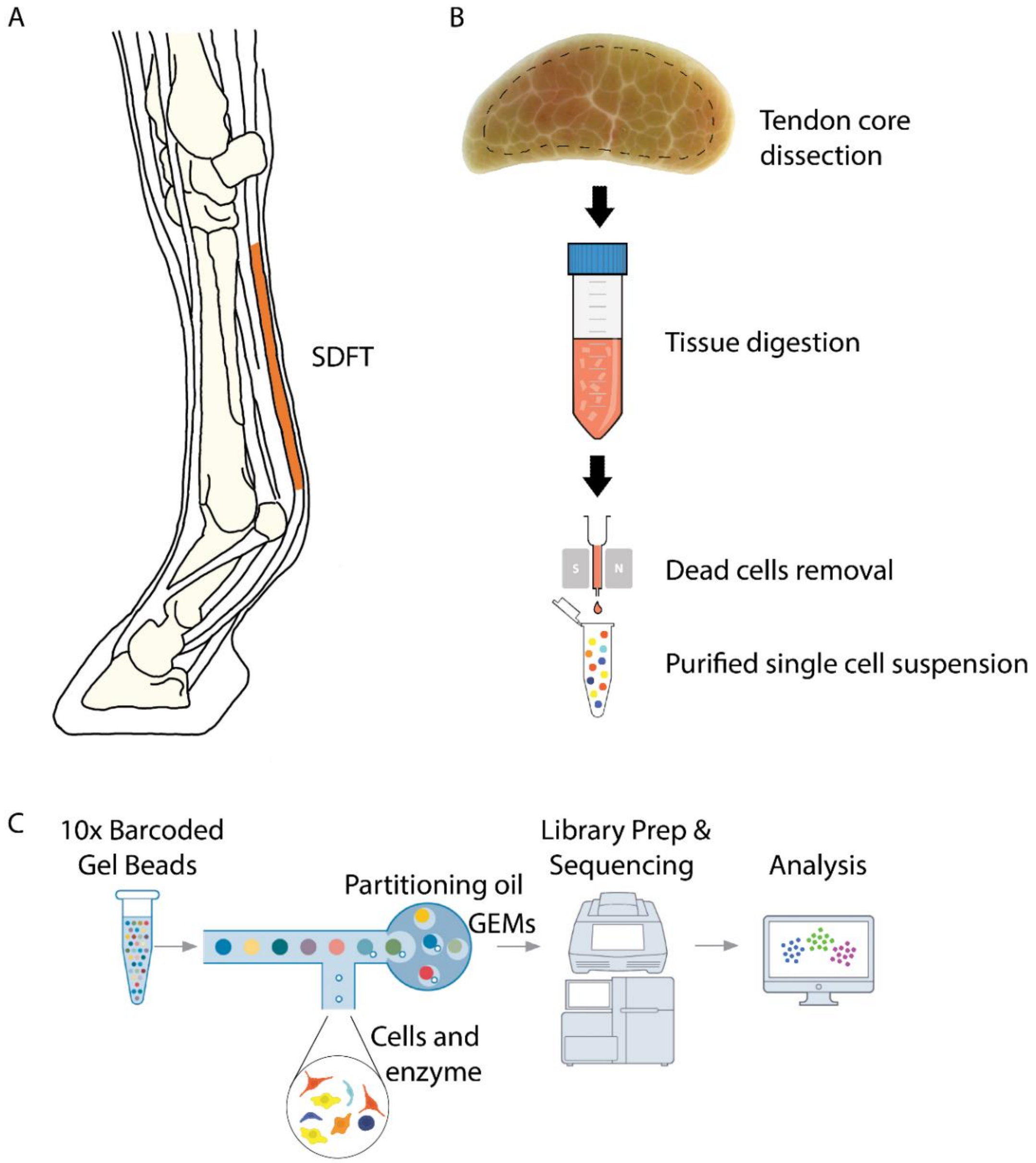

Supplemental Figure 1 (Related to Figure 1). Schematic showing sample collection and processing for single-cell RNA sequencing. (A) Superficial digital flexor tendons (SDFT) were harvested from the forelimbs of young and old horses (n=4/age group). (B) Samples were collected from the core of the mid-metacarpal region of the tendon, finely diced and enzymatically digested. Dead cells were removed to generate single cell suspensions. (C) Single cells were encapsulated using gel beads in emulsion (GEM), followed by library preparation, RNA sequencing and data analysis.

© 2023. Zamboulis DE et al. Published online at http://www.aginganddisease.org/EN/10.14336/AD.2023.0425-1

SUPPLEMENTARY DATA

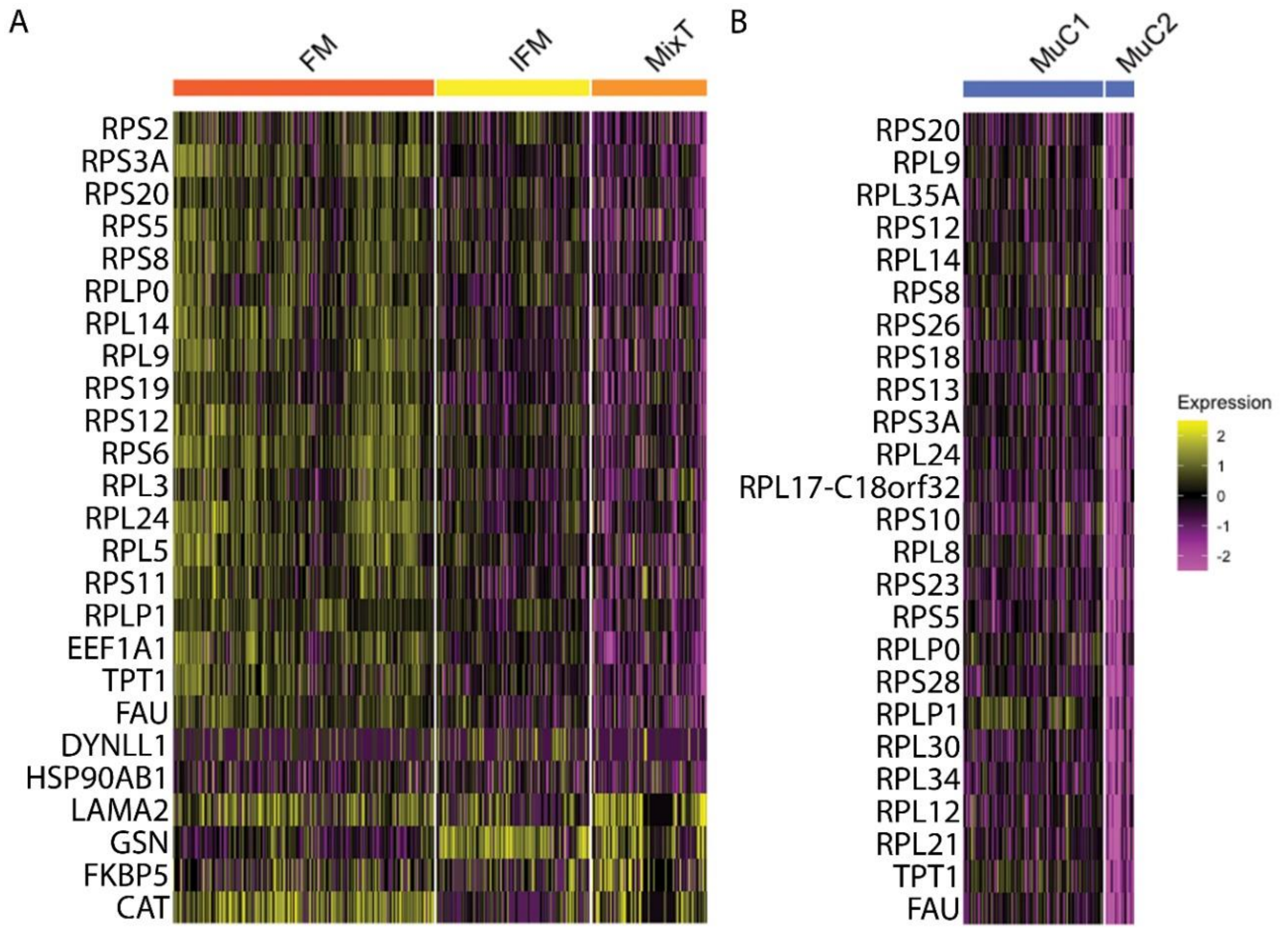

Supplemental Figure 2 (Related to Figure 1). (A) Heatmap depicting gene expression of the top 25 markers for the MixT cluster (ROC analysis) across the FM, IFM and MixT clusters (the ROC analysis returns a 'predictive power' ranked matrix of putative differentially expressed genes based on the ability of these genes to classify cells in a cluster). The MixT cluster shows lower expression of ribosome biogenesis-specific genes compared to the FM and IFM clusters. (B) Heatmap depicting gene expression of the top 25 markers for the MuC2 cluster (ROC analysis) across the MuC1 and MuC2 clusters. The MuC2 cluster shows lower expression of ribosome biogenesis-specific genes compared to the MuC1 cluster. Scale indicates log2FC expression and ranges from pink = <-2 to yellow = >2.

© 2023. Zamboulis DE et al. Published online at http://www.aginganddisease.org/EN/10.14336/AD.2023.0425-1

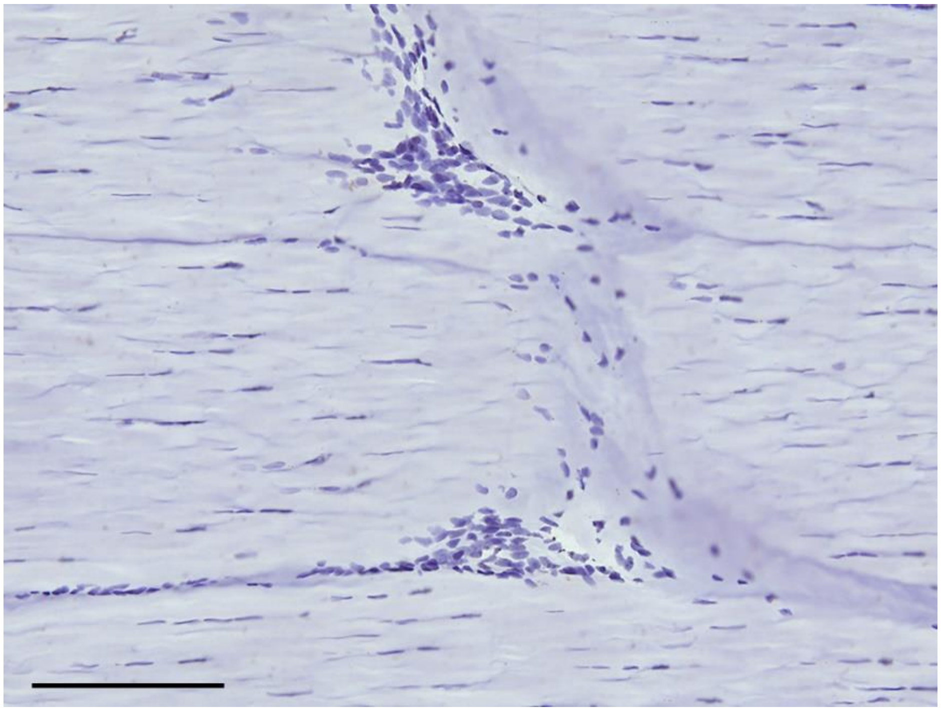

Supplemental Figure 3 (Related to Figure 1). Representative image of negative control. Negative control carried for the immunohistochemistry with omission of the primary antibody shows absence of DAB staining. Scale bar 75 µm.

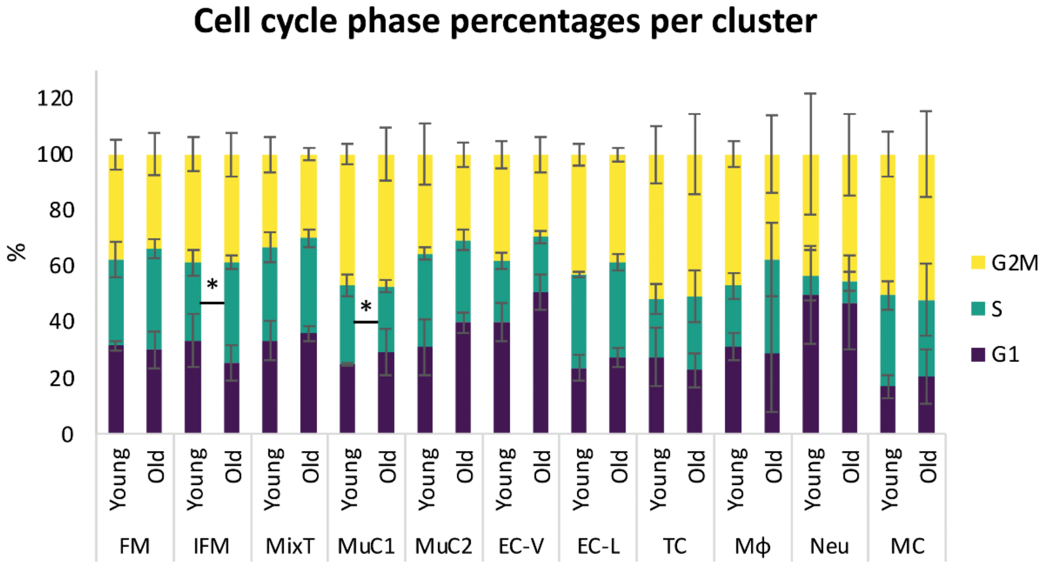

Supplemental Figure 4 (Related to Figure 2). Percentage of cells classifying as G1, S, and G2M phase per cluster in young and old samples (n=4/age group). The IFM tenocyte cluster and the MuC1 mural cell cluster were the only ones to show an age-related effect on cell cycling with a significantly larger percentage of cells classified in the S phase in old samples for the IFM tenocytes ($p=0.023$, unpaired t-test) whereas for the MuC1 cluster a significantly smaller percentage of cells classified in the S phase in old samples ($p=0.029$, Mann Whitney test).

© 2023. Zamboulis DE et al. Published online at http://www.aginganddisease.org/EN/10.14336/AD.2023.0425-1

SUPPLEMENTARY DATA

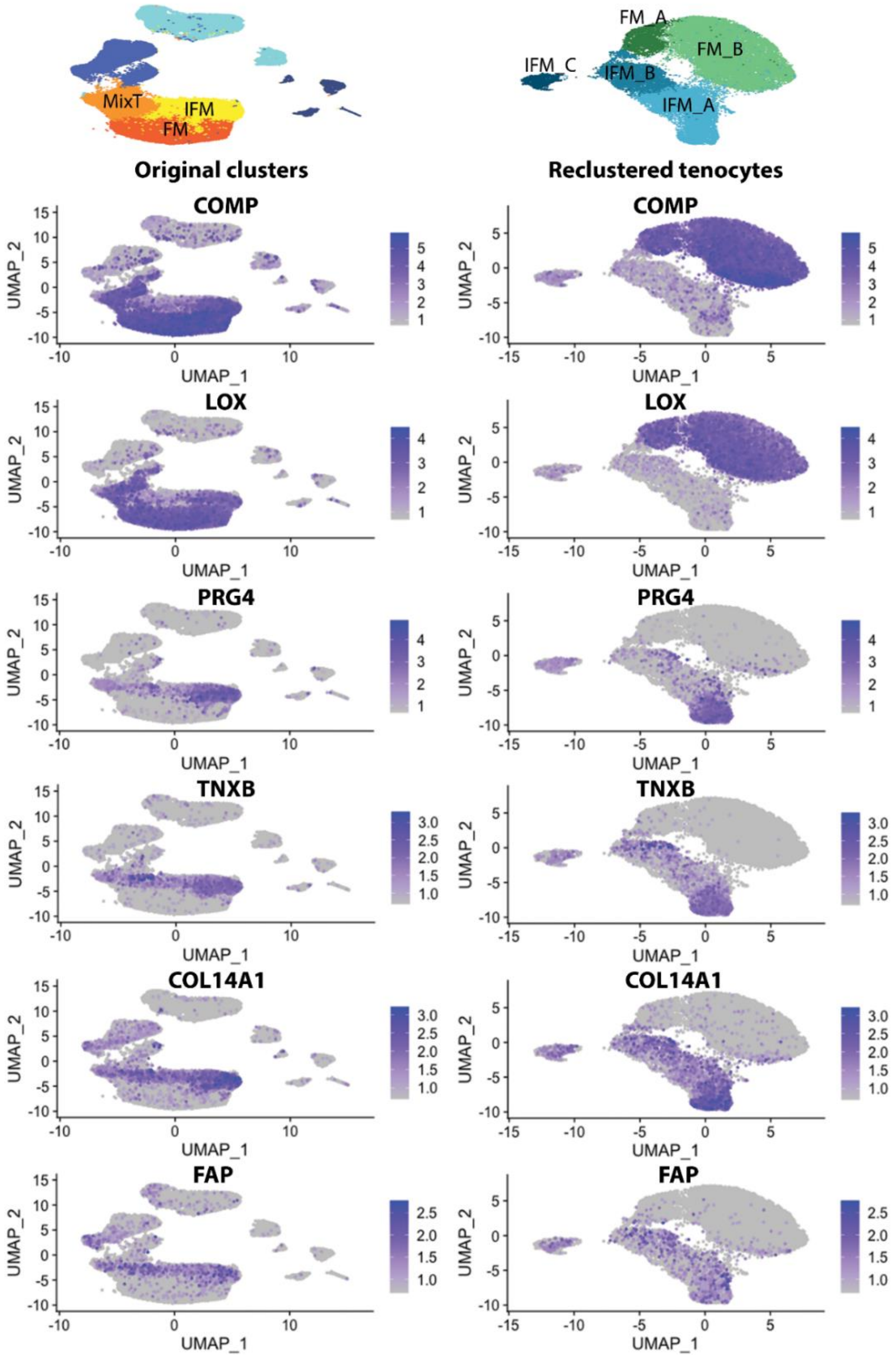

Supplemental Figure 5 (Related to Figure 3). UMAPs of *COMP*, *LOX*, *PRG4*, *TNXB*, *COL14A1* and *FAP* expression in the original tenocyte clusters and the re-clustered tenocytes. Tenocytes subclusters “FM_A” and “FM_B” and “IFM_A”, “IFM_B”, and “IFM_C” were confirmed as FM and IFM tenocytes respectively based on their differential expression of FM tenocytes markers *COMP* and *LOX*, and IFM tenocytes markers *PRG4*, *TNXB*, *COL14A1*, and *FAP*. Scale indicates expression and ranges from grey to blue.

© 2023. Zamboulis DE et al. Published online at http://www.aginganddisease.org/EN/10.14336/AD.2023.0425-1

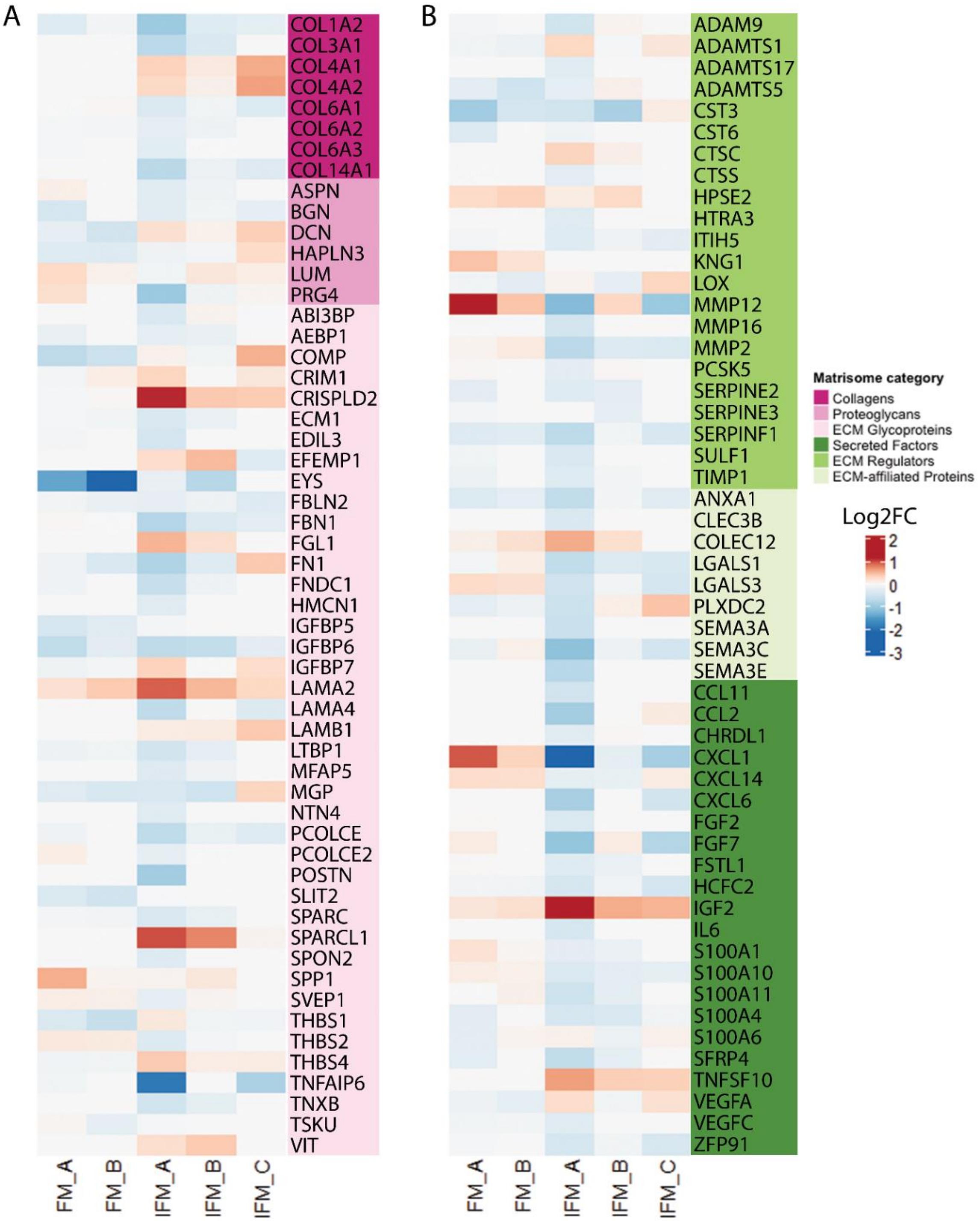

Supplemental Figure 6 (Related to Figure 4). Heatmap of DE core matrisome (n=4/age group) (A) and matrisome-related (B) genes with ageing in each tenocyte subcluster. The core matrisome categories, “Collagens”, “Proteoglycans”, and “ECM Glycoproteins”, and matrisome-related categories, “Secreted Factors”, “ECM Regulators” and “ECM-affiliated Proteins”, are colour-coded. Scale indicates log_2F2C and ranges from blue = –3, to white = 0, to red = 2.

© 2023. Zamboulis DE et al. Published online at http://www.aginganddisease.org/EN/10.14336/AD.2023.0425-1

SUPPLEMENTARY DATA

Supplemental Table 1. Details of primary and secondary antibodies, and blocking conditions used for immunolabelling experiments.

**Primary Antibody**	**Supplier**	**Dilution**	**Secondary Antibody**	**Supplier**	**Dilution**	**Blocking conditions**
LOX	Novus Biologicals (NB100-2530)	1:50	Goat anti-rabbit IgG	Dako	1:500	5% goat serum; 5% horse serum; 1% BSA
MYH11	Thermo Scientific (PA5-82526)	1:200	Goat anti-rabbit IgG	Dako	1:500	5% goat serum; 5% horse serum; 1% BSA
PECAM1	Abcam (ab28364)	1:50	Goat anti-rabbit IgG	Dako	1:500	5% goat serum; 5% horse serum; 1% BSA
TNXB	St John's Laboratory (STJ95967)	1:300	Goat anti-rabbit IgG	Dako	1:500	5% goat serum; 5% horse serum; 1% BSA
RGS5	St John's Laboratory (STJ95440)	1:600	Goat anti-rabbit IgG	Dako	1:500	5% goat serum; 5% horse serum; 1% BSA
CD74	St John's Laboratory (STJ96829)	1:100	Goat anti-rabbit IgG	Dako	1:500	5% goat serum; 5% horse serum; 1% BSA

© 2023. Zamboulis DE et al. Published online at http://www.aginganddisease.org/EN/10.14336/AD.2023.0425-1
